# Improved temporal resolution in ultrafast electron diffraction measurements through THz compression and time-stamping

**DOI:** 10.1063/4.0000230

**Published:** 2024-04-22

**Authors:** Mohamed A. K. Othman, Annika E. Gabriel, Emma C. Snively, Michael E. Kozina, Xiaozhe Shen, Fuhao Ji, Samantha Lewis, Stephen Weathersby, Praful Vasireddy, Duan Luo, Xijie Wang, Matthias C. Hoffmann, Emilio A. Nanni

**Affiliations:** SLAC National Accelerator Laboratory, Stanford University, Menlo Park, California 94025, USA

## Abstract

We present an experimental demonstration of ultrafast electron diffraction (UED) with THz-driven electron bunch compression and time-stamping that enables UED probes with improved temporal resolution. Through THz-driven longitudinal bunch compression, a compression factor of approximately four is achieved. Moreover, the time-of-arrival jitter between the compressed electron bunch and a pump laser pulse is suppressed by a factor of three. Simultaneously, the THz interaction imparts a transverse spatiotemporal correlation on the electron distribution, which we utilize to further enhance the precision of time-resolved UED measurements. We use this technique to probe single-crystal gold nanofilms and reveal transient oscillations in the THz near fields with a temporal resolution down to 50 fs. These oscillations were previously beyond reach in the absence of THz compression and time-stamping.

## INTRODUCTION

The ongoing quest in ultrafast science to reveal the fundamental principles of matter involves the use of x-ray free electron lasers (XFELs) and ultrafast electron diffraction (UED) to achieve femtosecond-scale temporal resolution,^1–7^ to visualize structural changes in processes ranging from photochemical reactions and lattice motion^8^ to phase transitions in gaseous, liquid, and solid-state matter.^9^ In particular, for UED, a time-resolved measurement can be made with electron probes from kilo-electron volt^10,11^ to mega-electron volt^2,7^ scales. For a given instrument, the UED temporal resolution is limited by the probe bunch length, and the timing jitter between the laser pump and electron probe. Leveraging bunch compression through achromatic magnets^12,13^ or using radio frequency velocity bunching^14,15^ can enable a temporal resolution on the order of tens of femtoseconds. Nevertheless, these aforementioned methods face facility constraints (e.g., additional length of beamline, additional rf sources or accelerating structures) and typically offer limited improvement in time-of-arrival jitter from radio frequency (rf) linacs. Taking advantage of a streak camera or time-dependent angular correlation to improve the temporal resolution has been previously investigated.^16–18^ However, the application of this method was so far limited to the kilo-electron volt scale with sub-picosecond temporal resolution.

Recent advances in THz acceleration have shown promise for manipulation of mega-electron volt scale electron bunches with high efficiency and small footprint.^19–24^ Previously, we have established a new technique for THz-driven pulse compression utilizing single and dual-fed THz compressor structures to enable much shorter electron pulses and simultaneously improve the time-of-arrival (TOA) jitter.^25^ In this article, we demonstrate improved temporal resolution in MeV-UED experiments with laser-driven THz radiation for velocity bunching and time-stamping compared to standard UED without bunch compression. Furthermore, we visualize femtosecond dynamics of THz time-stamped electron probes in UED measurements in single-crystal thin films to reveal transient near-field high-frequency EM oscillations relating to interaction of broadband THz radiation with such nanostructures.

### THz compression and time-stamping

**FIG. 1.**
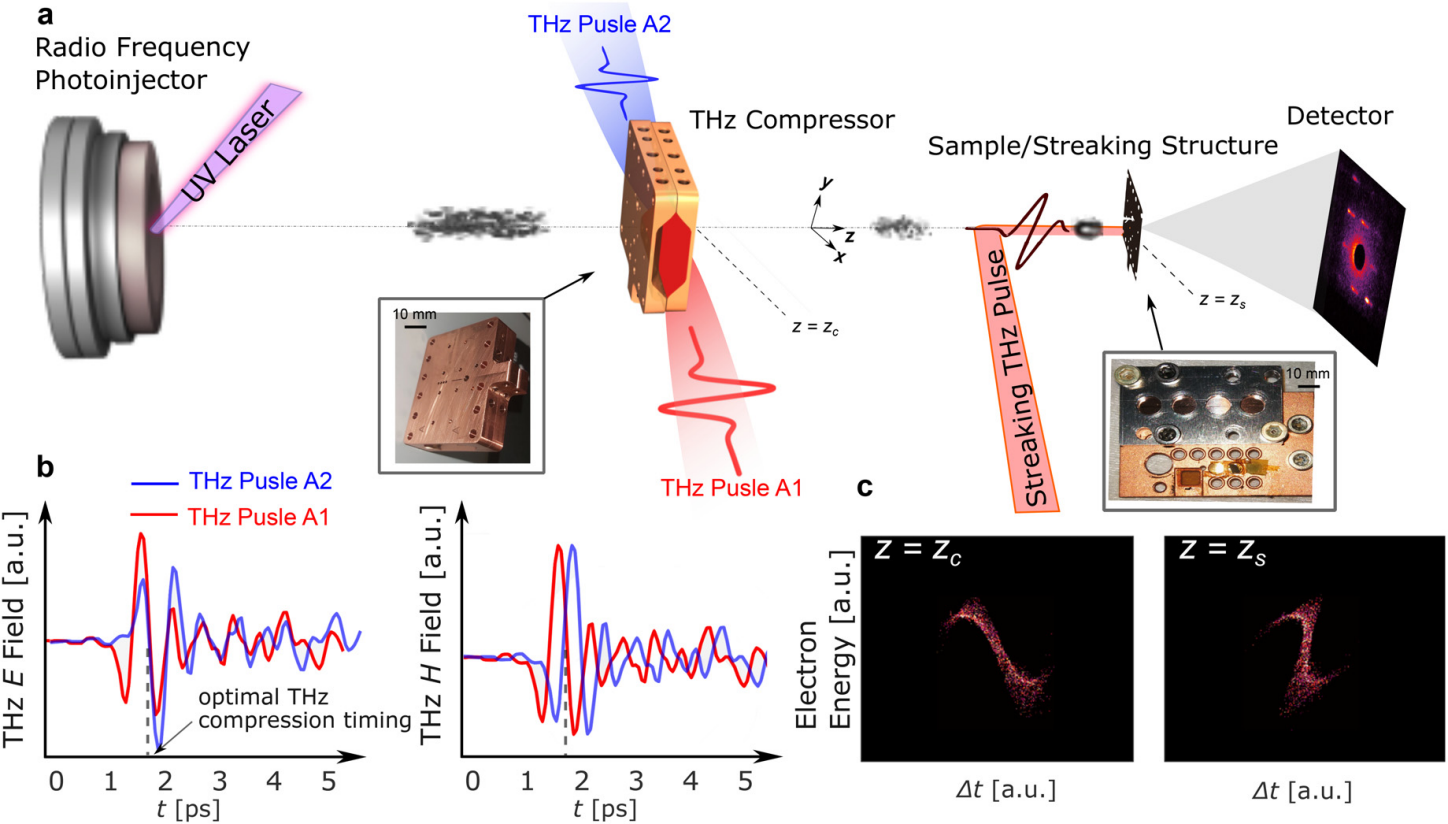
Schematic of the MeV-UED instrument employing dual-feed THz time-stamping. Single-shot electron probe interacts with two counter-propagating z-polarized THz pulses causing bunch compression at the sample 1 m downstream. The THz streaking pulse is polarized along the y direction. (b) Measured time profile of the THz electric and magnetic fields inside the THz compressor structure, which is shown in the inset. (c) Simulations of electron bunch spatial distribution at two different positions: *z_c_* is right downstream from the THz compressor, and *z_s_* is at the sample. Both the THz deflector (streaking) structure and the sample are mounted on the same stage shown in the inset.

We modified the MeV-UED beamline at SLAC National Accelerator Laboratory to allow for probing matter with an electron probe that has been compressed and time-stamped using terahertz radiation. The beamline schematic is shown in Fig. 1. 3 MeV, and 6 fC electron bunches are obtained from an rf photoinjector.^2,26^ The longitudinal compression and time-stamping of the relativistic electron bunch in the THz compressor with two counter-propagating quasi-single-cycle THz pulses centered at 1.7 THz.^25^ The parallel-plate THz structure provides dispersion-free focusing below the diffraction limit of the THz fields and strong field enhancement.^20^ The two THz pulses have the same electric field polarization with a relative delay of 200 fs to achieve the highest compression.^25^ The setup also includes a THz-driven streaking diagnostic^27^ mounted on the same stage as the sample. The THz pulse for streaking is obtained from a titled pulse front THz generation scheme.^28^

For compression, the ∼150 fs rms electron bunch enters the 100-*μ*m-diameter aperture in the THz structure, where the superposition of electric fields from the two THz pulses causes the energy of the trailing or delayed electrons to rise relative to the leading or early particles. This causes a longitudinal energy chirp of 100 ± 12 kV/ps for 1 *μ*J in each pulse. This is equivalent to a peak electric field seen by the electrons of 130 ± 15 MV/m.^25^ As a result of the difference in velocity of the electrons, the longitudinal profile of the electron bunch becomes compressed downstream, and temporal variation in the arrival time at the sample is suppressed (more discussion is found in Ref. 25). Figure 1(c) displays the simulated longitudinal energy distribution of the beam, illustrating the compressed bunch. Details of the data analysis technique and beam parameters are found in Appendixes A and B.

**FIG. 2.**
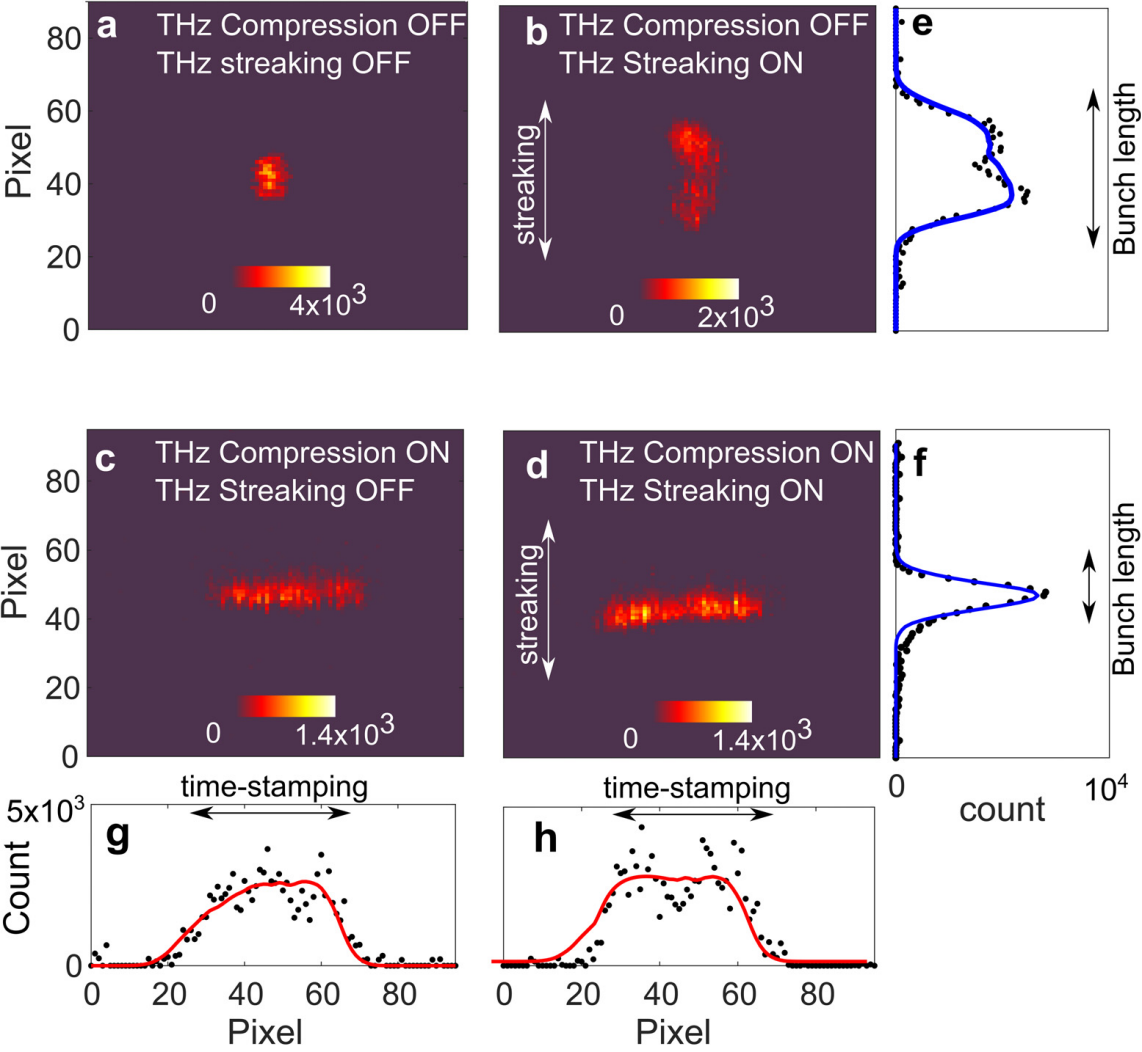
(a)–(h) Example single-shot images of the bunch in four different cases with and without THz compression and streaking, measured ∼3 m downstream from the THz compressor. The THz compressor stage adds an energy chirp for compression, and time-stamping along the x axis. The THz streaking maps longitudinal bunch distribution at the sample location onto the y axis (dotted lines represent data, while solid lines represent the fit). The compressed bunch in (c) and (d), which has a transverse size along the vertical axis of ∼5.5 pixel (0.55 mrad rms) and bunch length of 54 fs rms.

Since the two electric fields on the THz pulses have the same polarization at the interaction phase, the transverse magnetic fields add up causing a time-dependent deflection, see Fig. 1(b). Based on the measurement of the electron bunch length and jitter, a minimum electron probe temporal length is measured to be 40 ± 9 fs rms [FWHM 94 fs] down from an average of 154 ± 37 fs rms [FWHM 367.2 fs].^25^ A simultaneous improvement of the bunch's shot-to-shot time of arrival (TOA) is achieved, with a minimum TOA jitter of 23 fs rms, down from 69 fs rms. The resulting bunch distributions at the detector ∼2 m downstream from the sample are depicted in Fig. 2 showing a single shot of the electron distribution, with the background subtracted. These images taken when THz compression is enabled represent a time-stamping that has been imparted on the transverse bunch profile from the THz interaction (see Appendixes A and B).

### Improved time resolution of UED with THz time-stamping

Thanks to THz compression, electrons in each bunch are time-stamped with a correlated time and position distribution, as shown in Fig. 2(c). The electron bunch image from Fig. 2(d) is analyzed to correlate the transverse deflection from the compressor with the arrival time of the electrons, and this is shown in Appendix C (Fig. 5). Each vertical slice corresponds to a different time-of-arrival of the time-stamped bunch.

Here, we utilize this time-stamping feature to demonstrate improved the temporal resolution of UED measurement by examining the response time and the transient dynamics of pump–probe measurements in a 11-nm-thick Au(100) film. The vertically polarized, broadband THz (streaking) pulse with peak electric field 20 MV/m produces a fluence of 50 *μ*J/cm^2^ which is used to excite the Au nanostructure. The pulse central frequency 0.7 THz and FWHM of 0.45 THz. Simulations of the THz interaction with the sample show transient oscillations occurring at frequencies beyond 1 THz as seen from the spectrum of the electric field in Fig. 4(c) (see Appendix D for more details). These plasmonic resonances result in transverse deflections of the diffracted electrons from the local electromagnetic fields. Our measurements utilize the time-dependent integrated intensity and centroid variations of the (220) Bragg peak in Au.^29^ Note that Bragg peak intensity variations are not a direct result from the plasmonic resonance, but are attributed to the difference in angle of incidence of the beam on the sample from shot to shot.^30^ All Bragg peaks exhibit the same time-dependent behavior.

**FIG. 3.**
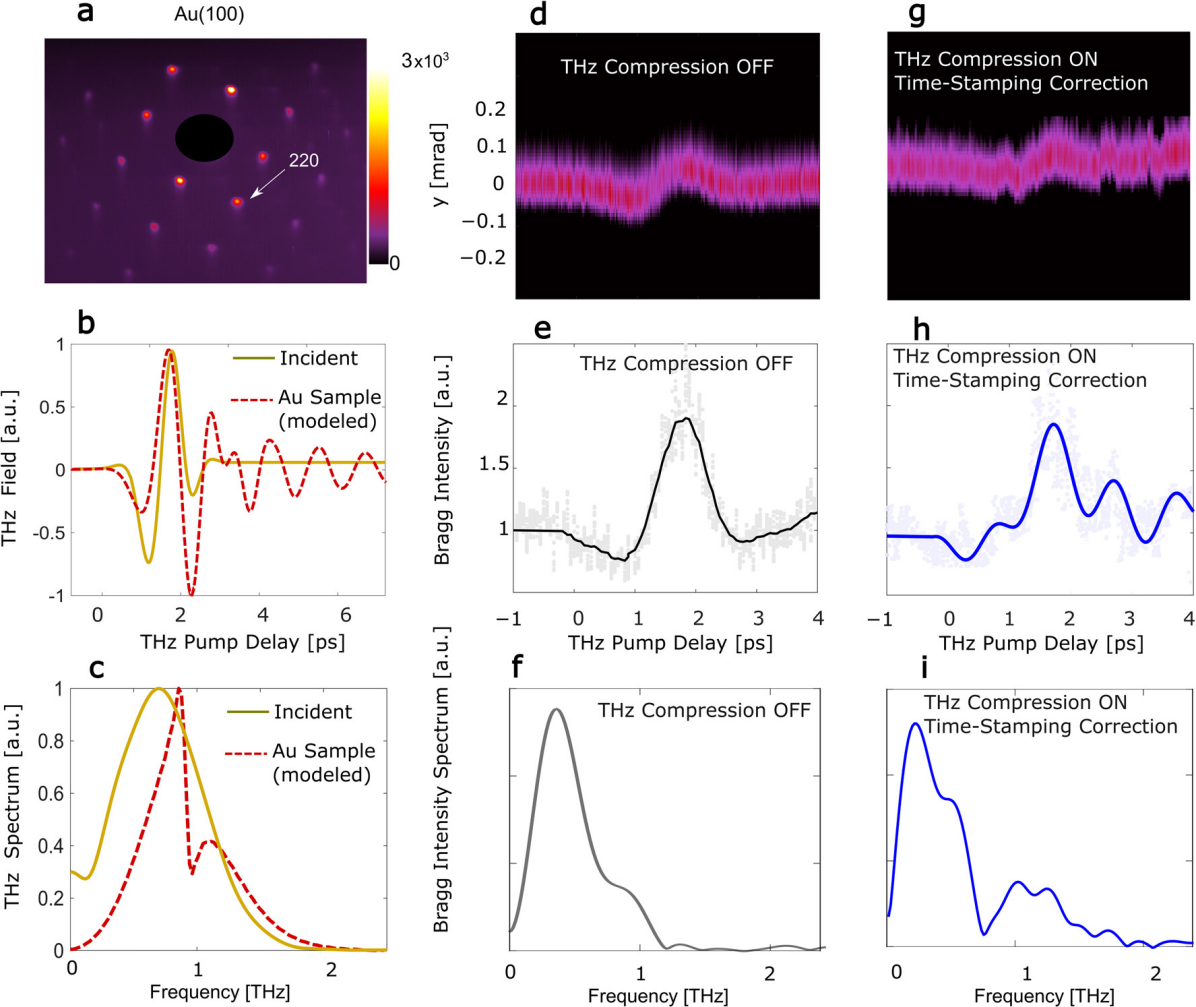
Temporal resolution of THz compressed and time-stamped single-shot UED probes with single-crystal Au sample. (a) Diffraction pattern of Au(100) sample obtained from integrating 1000 shots, (b) THz pump field waveform generated from a LN setup and the corresponding waveform simulated based on interaction with the sample, (c) the spectrum of these THz waveforms in (b). (d) Measured time-dependent sliced beam transverse deflection along the y axis of the (220) Bragg peak of the single-crystal Au film as a function of the THz pump delay without THz compression, and (g) with THz compression and time-stamping correction showing a faster response time. (e)–(h) The corresponding normalized integrated intensity of the Bragg peak on a single-shot basis (symbols) and averaged (lines), and (f)–(i) the Fourier transform of the integrated intensities in (g) and (h) with the average subtracted, showing the transient oscillations in the dynamical Bragg intensities for THz compression with time-stamping correction around 1.1 THz.

**FIG. 4.**
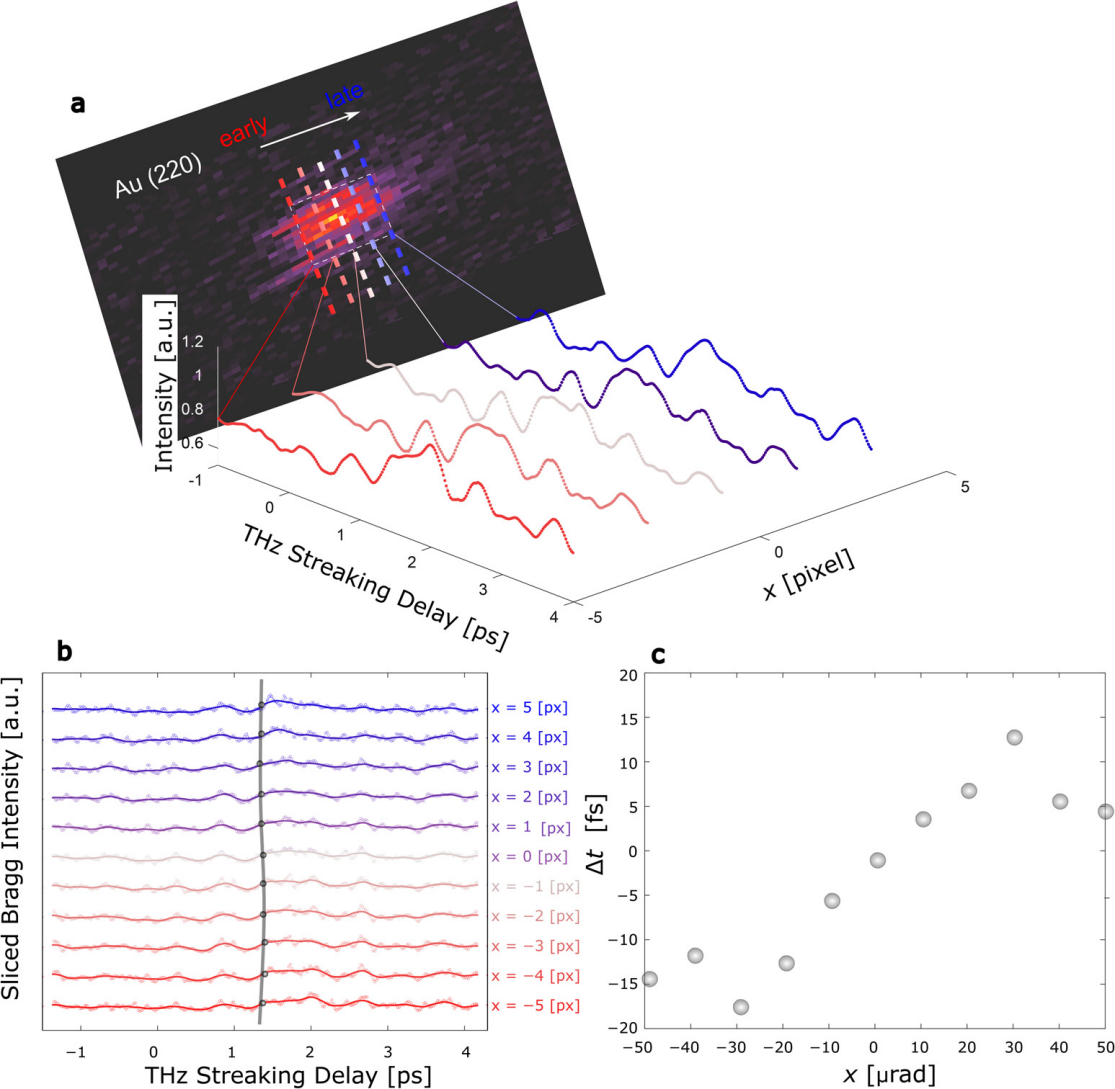
Time-stamping of single-shot UED probes in THz-excited single-crystal Au. (a) Stacked sliced Bragg spot intensities (rainfall plot) are taken on a per-pixel basis of the transverse beam distribution along x, with variation as a function of the THz pump delay. (b) The location of the maximum upward slope of the sliced intensity is designated by the marker and shown as a function of sliced angular location showing an accuracy in temporal measurement of ∼0.43 fs·*μ*rad^−1^ in the linear region |x| < 20 *μ*rad, beyond which the beam exhibits a nonlinear pileup from the THz compression interaction. Note that intensity data beyond the Bragg peak spot size of ∼48 *μ*rad exhibits high fluctuations due to reduced counts.

**FIG. 5.**
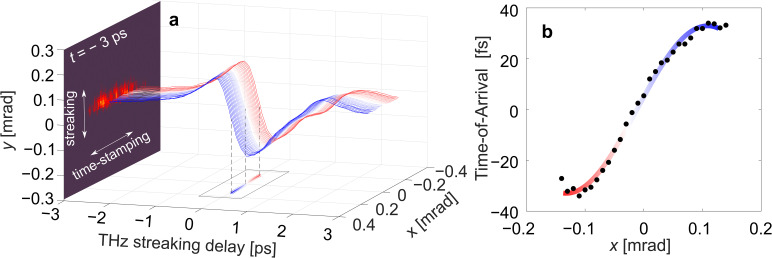
THz streaking of time-stamped electron bunch. This measurement is used to find the calibration accuracy in measuring the temporal resolution, which yields 4.5 fs/pixel or 0.45 fs/*μ*rad, as seen from the slope in (b).

In Fig. 3, we show a comparison between no THz compression and with THz compression aided by the time-stamping correction. In the latter, the time-stamp is used *a posteriori* to correct the TOA jitter at the sample, using the algorithm in (Appendix C). The time-dependent (220) Bragg spot transverse deflection (along the y axis) in addition to the normalized integrated intensity due to the THz pump is depicted in Figs. 4(d), 4(e), 4(g), and 4(h), obtained on a single-shot basis over 5 ps. The normalized amplitude spectra through the Fourier transform of the integrated intensities with average subtracted are also shown Figs. 3(f) and 3(i). The Bragg peak intensities exhibit fast oscillations seen in the intensity spectrum when time-stamping correction is applied. We attribute these oscillations to resonances in the gold sample at frequencies >1 THz, which cannot be resolved as evident from the uncompressed case in Figs. 3(d)–3(f). We have confirmed these observations through a full electromagnetic beam dynamics model, see more details in Appendix D.

While the previous analysis included integration of counts across multiple discrete electron bunches, the time-stamping technique can also be utilized for single-shot measurements, as shown in Fig. 4. In Fig. 4, the single-shot time-stamping dynamics are shown with the Bragg peak intensity variations as a function of the THz streaking delay. Each pixel slice of the time-stamped image shows a similar transient response to the integrated beam intensity but exhibits a delay corresponding to the time-of-arrival of each slice with respect to the THz pump. The time-stamping resolution, defined as the linear slope of the time-of-arrival as a function of transverse position on the detector, is 5/fs/pixel or ∼0.43 fs *μ*rad^−1^ accounting for the geometrical factor of our experiment. This means that the minimum temporal resolution that we are measuring on a single pixel of the detector is 5 fs. This angle is dictated by the THz arrival time, which can be corrected for using the time-stamp but causes intensity variations in each Bragg peak.

The time-stamping exhibits nonlinearity due to the pileup from the long initial electron probe length during the compressor interaction as seen in Fig. 4(c). Indeed, the linear proration of the single-cycle THz field in the compressor structure only extends up to 170 fs providing a linear time-stamping within ±73 *μ*rad. Finally, we stress that the minimum temporal resolution that can be realized from this time-stamping scheme is limited by the spatial resolution of the detector as well as the overall THz energy in the compressor stage. Though we have used the time-stamping features primarily for single-crystal samples, it can be readily adapted to perform measurements in polycrystalline samples, see Appendix E.

## CONCLUSION

In summary, we have demonstrated electron compression and time-stamping to produce ultrashort electron probes for MeV-UED. These electron probes were used to demonstrate improved temporal resolution for observing diffraction dynamics in crystalline materials. This time-stamping approach offers a pixel-by-pixel representation of temporal dynamics visible on the detector, thereby enhancing the observable resolution. Additionally, this method allows for further improvement in temporal resolution by optimizing the shape of the THz pulse and preconditioning the UV drive laser to generate shorter initial probes.

Advanced techniques in THz compression and time-stamping correction can be developed for high charge UED beamlines through more tailored THz interaction. This is done by improving the efficiency of terahertz sources and optimizing the structure to enhance interaction impedance. Finally, a detailed study of THz-pump-UED probe^29^ in single-crystal samples can provide further understanding of carrier dynamics, strong photofield emission, and plasmon interactions,^29^ as well as carrier oscillations,^31,32^ in previously inaccessible fs time scales. The time-stamping method showcased here is applicable even in standard UED operation with a pump laser, without the need for THz compression or THz pumping. In this scenario, a THz time-stamping component can be placed downstream of the diffraction detector,^33^ offering comparable spatiotemporal correlation and jitter correction capabilities without affecting the UED measurements.

## Figures and Tables

**FIG. 6.**
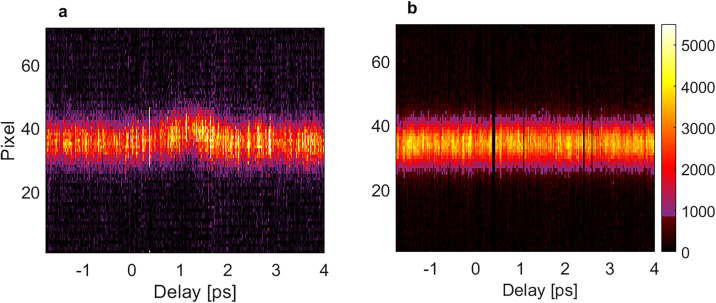
Beam distribution along the x direction of the Au (220) Bragg peak. (a) Before time-stamping and (b) after time-stamping. The time-stamping formula used in this plot is $t_{\text{TOA}}=t_{s}-D(x_{s}-x_{r})$ given *D* = 4.5 fs/pixel. The small jump in the beam distribution in (a) is possibly due to an undesired x-component in the THz pump electric field.

**FIG. 7.**
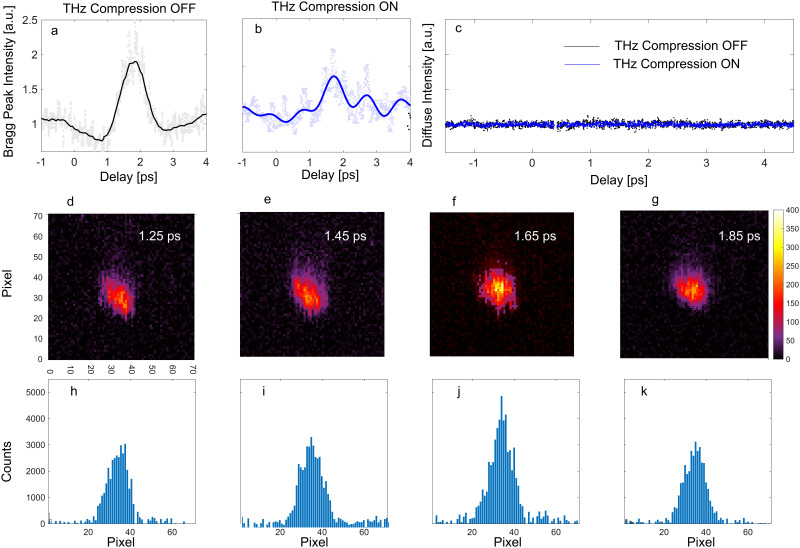
Results of time-stamping of electron probes in THz pumped UED probed Au sample with time-stamping. The Bragg peak (220) intensity of the full beam in (a) and (b) shows a clear observation of fast dynamical resonance corresponding to a plasmonic surface mode. (d) The diffuse background intensity of the Bragg peak shows minimal fluctuations <3%. (d)–(g) Beam distributions of the (220) Bragg peaks as a function of time delay as well as (h)–(k) the corresponding beam distributions showing the fast dynamical response and increase in counts within 200 fs.

**FIG. 8.**
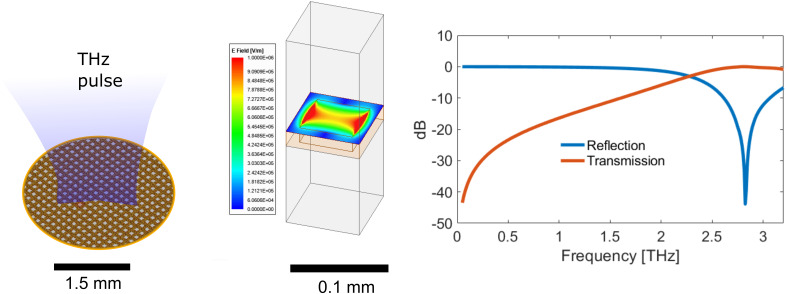
Simulation of THz field interaction with an Au thin film on top of a TEM grid. The electric field profile at the grid resonance ∼2.7 THz is also shown. The reflection and transmission of the grid show a frequency dependent response, which also indicates that the THz pulse experiences dispersion at frequencies covering the incident THz pulse up to 2 THz.

**FIG. 9.**
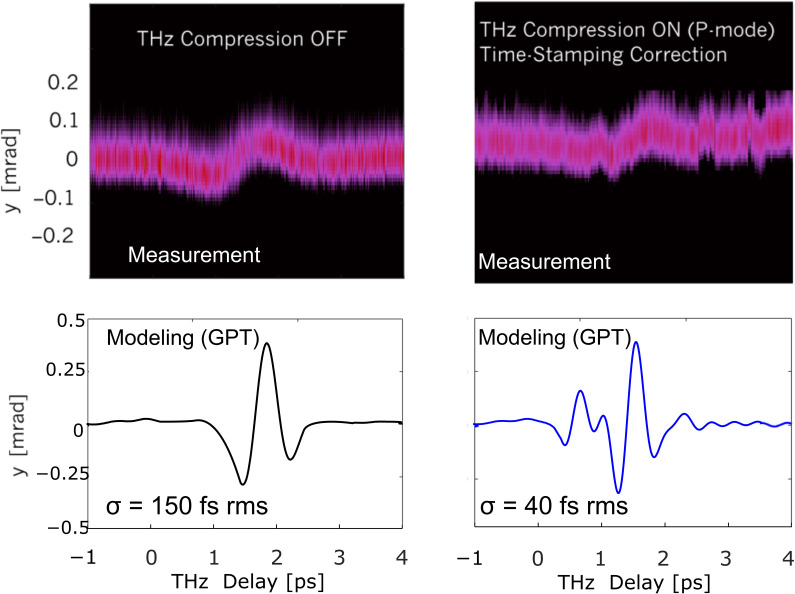
Comparison between the measured THz streaking of the Au Bragg spot and the corresponding GPT model for THz streaking from an Au film on top of a TEM grid showing that the GPT model can predict the ringing oscillation in the time-dependent transverse deflection when a small probe length is used (in the compressed), whereas these features are smoothed out for the longer probe (uncompressed).

**FIG. 10.**
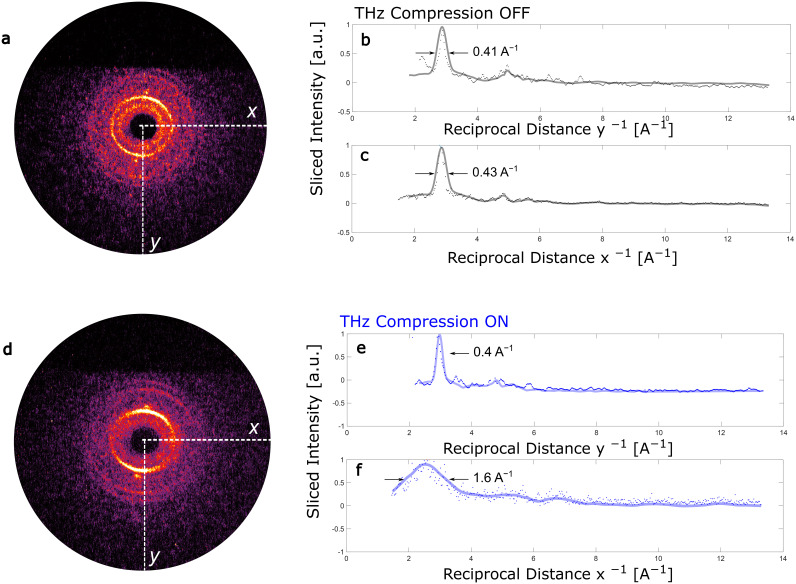
Comparison between the diffraction patterns obtained from MeV-UED in two cases: (a)–(c) THz compression off, (d)–(f) THz compression. Broadening of the Bragg peak is evident in the sliced intensity along the x-direction in the time-stamping mode compared to the other cases, while the Bragg peak remains almost the same in a perpendicular cut (along y). For comparison purposes, the diffraction patterns were measured using the ∼4 fC bunch and averaging about 36 single-shot images.

## Data Availability

The data that support the findings of this study are available from the corresponding author upon reasonable request.

## APPENDIX A: ANALYSIS OF BEAM IMAGES

Individual single-shot beam image data collected from the electron-multiplying charged coupled devices (EMCCD) camera were analyzed and fitted to a two-Gaussian model. To measure the electron probe length and TOA from the THz streaking setup, we used a femtosecond per pixel conversion obtained by scanning the probe centroid as a function of the THz streaking delay. Our method also corrects the “pileup” at the ends of the projected distribution and maps it to a position-dependent fs/pixel conversion. All images that did not pass a cutoff in the confidence interval of the probe length fit with the Gaussian distribution were removed from the final calculation, which only occurred in the original beam probes with no THz compression. An additional filter was applied that removed all data with a compressor time of arrival greater than ±100 fs from the minimum probe length and compression factor calculation. This reduced some of the effects of beam jitter in the result so that the minimum probe length value was only calculated from shots that had arrived at the compressor within a reasonable time frame. The standard deviation for the probe compression factor was calculated using standard error propagation.

## APPENDIX B: BEAM PARAMETERS

The original UED electron bunch probe used in our experiment is estimated (based on observed diffraction patterns and simulations) to have the following parameters: initial energy spread of <2 keV rms, normalized emittance of 8 nm-rad, and pointing jitter of about 50 *μ*rad. After THz compression, the pointing jitter is about 70 *μ*rad in the y direction, and simulations have shown that the energy spread is about 3.3 keV rms. The major contributor of energy spread degradation is slice energy spread growth, which has been observed up to 2 keV rms, even as the overall energy spread is about 3.3 keV rms. The normalized emittance from THz interaction has increased from 8 nm-rad rms in both x–x and y–y′ phase spaces, to 105 nm-rad rms in the x–x′ phase space, and 20 nm-rad rms at the sample. Though the spatial spread of the beam in the x axis can obscure some details in diffraction from samples requiring fine momentum resolution, it can be corrected by focusing quadrupole magnets. The charge is about 6 fC with a shot-to-shot fluctuation of <10%.

## APPENDIX C: TIME-STAMPING ALGORITHM FOR CRYSTALLINE SAMPLES

The time-stamping correction algorithm utilized in this work uses the transverse beam (Bragg peak) distribution along the x direction (in which the THz compressor induces spatiotemporal correlation) to correct for the time-of-arrival jitter of the probe with respect to the THz pump at the sample. Figure 5 shows that the bunch that has a transverse size of ∼5.5 pixel (0.55 mrad rms) has an overall bunch length of 54 fs rms. The bunch distribution along the x direction corresponds to TOA at the sample and may be used in post-processing as a time-stamp to increase the temporal resolution below the overall electron bunch length.

For diffraction patterns obtained from a single-crystal Au sample, beam images were taken at different stage positions, *t_s_*, using a 50 fs step, with 20 images collected at each delay stage position. The actual time-of-arrival *t*_TOA_ of every single shot can then be obtained by

$$t_{\text{TOA}}=t_{s}-D(x_{s}-x_{r}),$$
(C1)

where *D* is the calibration of the THz-induced time-stamping from the compressor interaction, *x*_s_ is the transverse centroid of the beam, and *x_r_* is the calibrated transverse centroid taken from a reference scan (with THz compression off). After evaluating *t*_TOA_ for each shot and correcting for the jitter, we average shots with TOAs within ±20 fs to provide better statistics of beam distribution. The beam intensity is then calculated per pixel in the image as in Fig. 4. In our model, we used *D* = 4.5 fs/pixel. We also note in the results in Figs. 6 and 7 that the background diffuse intensity fluctuations is ∼3%, which is responsible for beam charge variation shot to shot. However, these fluctuations do not impact the Bragg peak intensity. Figure 6 also shows the counts before and after the time-stamping correction and the 20 fs time binning; and the results in Figs. 3 and 4 are based on this correction.

## APPENDIX D: THZ NEAR-FIELD INTERACTION WITH AU NANOSTRUCTURE

Ultrafast pulses can excite energetic electrons in Au film, in the timescale of tens of femtoseconds through electron–electron collisions, until they thermalize to the lattice at the picosecond timescale.^34^ Though strong THz electric field may enable electron photofield emission from the Au film, the THz streaking field used in our study has a very low energy fluence. Accordingly, the energy deposited by the THz radiation on the film induces a negligible ultrafast dynamics related to electron motion in the crystal. We argue that the measurable Bragg intensity variations are due to the THz near fields close to the surface of the gold structure that streak these Bragg peaks.

In addition, we point out that Mohler *et al.*^11^ have shown that all-optically generated single electron pulses can capture dynamic EM interactions in a nanostructures with time resolution of few femtoseconds. In such case, the modulation of the electron probe quantum mechanical phase front is observed on the diffraction pattern and could be used to reveal the same electromagnetic dynamics with nanometer and sub-light-cycle precision. However, we argue that this only applies to the case of single electrons where quantum mechanical interference can occur. In our study, the UED bunches have a measurable energy spread, and their size not too small compared to the wavelength of THz radiation (~170 μm), we resort to classical justification of the oscillations observed in Fig. 3 through an EM dynamic model.

We have performed EM simulations using Ansys HFSS^35^ and Lumerical^36^ of the thin Au film supported by a TEM grid and excited by a THz single-cycle pulse. The gold TEM grid is 20 *μ*m thick and has pitch of *p* = 90 *μ*m with square apertures of sides *w* = 65 *μ*m. To model the electromagnetic interaction of the THz pulse with the grid, we use Ansys HFSS (Fig. 8) assuming a THz Gaussian beam impending on the structured grid with the Au film. Because the THz pump beam spot size (FWHM ∼2.5 mm) on the grid is much larger than the aperture size, we can treat the grid as a periodic structure. We ignore in this model the nonlinear time-dependent conductivity change due to ultrafast lattice heating in the thin Au film induced by THz excitation since the THz field is not sufficiently high to raise the electronic temperature in the Au sample. The presence of the grid causes frequency dispersion of the incident broadband pulse thanks to the phase variation of waves at Au sample interface at different frequency components. The Au dielectric properties are obtained from Ref. 37, and the transient simulations were also verified in Ansys Lumerical. In Fig. 8, we show the transmission and reflection frequency spectrum resulting from THz interaction with the Au sample and grid using HFSS, confirming the dispersive characteristics of the grid, as well as a transmission resonance occurring at a higher frequency ∼2.7 THz.

Based on these modeled dynamical fields, we performed GPT (General Particle Tracer^38^) simulations of the electron probe dynamical interaction with the system assuming that the main beam is fully transmitted through the Au film (our model does not account for diffraction). These simulations were performed by including frequency components of the THz pulse up to 2 THz and assuming a probe length of both 40 fs and 150 fs (uncompressed) to mimic the measurement result. The streaking curves for both cases are shown in Figs. 9 and 4 in the paper, indicating that indeed the longer bunch cannot resolve the fast THz oscillation dynamics occurring due to the dispersive interaction with the grid system.

## APPENDIX E: TIME-STAMPING ALGORITHM FOR POLYCRYSTALLINE SAMPLES

For polycrystalline samples, the ring diffraction pattern makes it challenging to characterize the time-stamping temporal resolution. However, we show in Fig. 10 that it is possible in principle to measure the broadening of the Bragg ring in the diffraction pattern of Bi with time-stamping. This is done by slicing the diffraction pattern along the direction of THz-induced time-stamping. We see that the Bragg ring at s = 2.84 A^−1^ has been broadened by the time-stamping probe beam from having a FWHM of 0.43 to ∼1.6 A^−1^ only due to the transverse distributing of the beam in the time-stamping direction; while not impacting the Bragg peak size in the perpendicular direction. Note that a rigorous convolution analysis will be required taking into account the beam spatial distribution, emittance, and time-stamping correction to obtain the temporal resolution.

## APPENDIX F: SAMPLE INFORMATION

The THz compressor structure was fabricated from OFE copper.^25^ The structure was then assembled from six individual parts to obtain a precise beam tunnel (radius of 45 *μ*m). The minimum gap between the parallel plates is 75 *μ*m. The structure is bolted together and mounted inside the vacuum chamber with a 3-axis motorized stage for coupling optimization of the THz pulses.^25^ The Bi sample is a 35-nm-thin film grown on free-standing silicon nitride (Si_3_N_4_) membranes using molecular beam epitaxy (MBE) in the (100) orientation. The Au sample was obtained from Ted Pella,^39^ and it is a large area single-crystal gold film, approximately 11 nm thick grown in the (100) orientation and suspended on a 3-mm-diameter gold TEM grid of thickness 20 *μ*m. The TEM grid with the Au film supports transient oscillations at THz frequencies through THz interaction with the film and the periodic TEM grid.
